# Web-Based, Interactive, Interest-Based Negotiation Training for Managing Conflict in Isolated Environments: Opportunistic Study With an e-Survey

**DOI:** 10.2196/42214

**Published:** 2023-06-09

**Authors:** Jennifer Fleischer, Jeff Ayton, Maree Riley, Kim Binsted, Devin R Cowan, Abigail M Fellows, Jeff A Weiss, Jay C Buckey

**Affiliations:** 1 Space Medicine Innovations Laboratory Geisel School of Medicine at Dartmouth Lebanon, NH United States; 2 Australian Antarctic Division Kingston Australia; 3 University of Hawaii Honolulu, HI United States; 4 Mass General Brigham Boston, MA United States

**Keywords:** conflict management, bargaining, confined environments, COVID-19, pandemic, development, environment, skill, training, users, essential, Australia, management

## Abstract

**Background:**

Effective negotiation in relationships is critical for successful long-duration space missions; inadequate conflict resolution has shown serious consequences. Less desirable forms of negotiation, including positional bargaining (eg, negotiating prices), can exacerbate conflicts. Traditional positional bargaining may work for simple, low-stakes transactions but does not prioritize ongoing relationships. High-stakes situations warrant interest-based negotiation, where parties with competing interests or goals collaborate in a mutually beneficial agreement. This is learnable but must be practiced. Refresher training during conflicts is important to prevent out-of-practice crew members from using less effective negotiation techniques. Training should be self-directed and not involve others because, on a space mission, the only other people available may be part of the conflict.

**Objective:**

We aimed to develop and test an interactive module teaching principles and skills of interest-based negotiation in a way that users find acceptable, valuable for learning, and enjoyable.

**Methods:**

Using a web-based, interactive-media approach, we scripted, filmed, and programmed an interest-based negotiation interactive training module. In the module, the program mentor introduces users to “The Circle of Value” approach to negotiation and highlights its key concepts through interactive scenarios requiring users to make selections at specific decision points. Each selection prompts feedback designed to reinforce a teaching point or highlight a particular negotiation technique. To evaluate the module, we sought populations experiencing isolation and confinement (an opportunistic design). This included 9 participants in isolated, confined environments in the Australian Antarctic Program and the Hawai'i Space Exploration Analog and Simulation Mars simulation, as well as a subset of people who self-identified as being isolated and confined during the COVID-19 pandemic. Feedback was collected from participants (n=54) through free-response answers and questionnaires with numerical scaling (0=strongly disagree to 4=strongly agree) at the end of the module.

**Results:**

In total, 51 of 54 (94%) participants found the activity valuable for learning about conflict management (identified by those who selected either “somewhat agree” or “strongly agree”), including 100% of participants in the isolated and confined environment subset (mode=3). In total, 79% (128/162) of participant responses indicated that the module was realistic (mode=3), including 85% (23/27) of responses from participants in isolated and confined environments (mode=3). Most participants felt that this would be particularly valuable for new team members in an isolated, confined environment (46/54, 85% of all participants, mode 4; 7/9, 78% of the isolated and confined environment subset, mode 3) as well as veterans.

**Conclusions:**

This module offers a self-directed, consistent approach to interest-based negotiation training, which is well received by users. Although the data are limited due to the opportunistic study design, the module could be useful for individuals in isolated and confined environments and for anyone involved in high-stakes negotiations where sustaining relationships is essential.

## Introduction

Effective teamwork, both within and among parties, is widely recognized as a core component of any successful organization or exploration campaign [1]. This is particularly true in isolated and confined environments (ICEs) such as remote Antarctic research bases, submarines, and space missions, where trust and positive interpersonal relations are paramount [1-5]. Effective teamwork does not mean the absence of disparate opinions or conflict; rather it is the ability to navigate these situations successfully [1,2].

Multiple documented incidences of conflict have occurred in the space program, with varying consequences [2-8]. On Apollo 7, conflict between the commander and ground control was evident through criticism and disrespectful comments in communications. Although this did not lead to any direct mission consequences, and the mission was the commander’s last flight, none of the other crew members were assigned to other missions. In the Russian Mir 23 space station mission not only did strained relations between the crew and ground control result in shouting over the air-to-ground loop, but the lack of effective interpersonal communication is also regarded as a contributing factor in the collision of an unmanned Progress module into the station [4]. Interpersonal conflicts have been cited as being responsible for the shortening of the missions Soyuz 21, Soyuz T-14, and Soyuz TM-2 [5]. On the Skylab 4 mission, conflict surrounding crew schedules led to such tension that the crew ultimately cut off communications in what has been referred to as the Skylab “Mutiny” [4,6].

ICEs can, by their very nature, exacerbate the disagreements, discordant opinions, and conflict that inevitably arise in group settings [2,3,6,9,10]. The importance of being able to manage these situations effectively cannot be overstated. Although medical factors (eg, appendicitis) are often viewed as potential reasons to terminate space missions, interpersonal conflicts on long-duration missions may present more of a risk of mission termination than any medical or physiological factor [4]. The recent COVID-19 pandemic, with recommendations for social distancing and self-quarantine, expanded the number of people experiencing ICEs and provided many with personal experience highlighting how challenging navigating these situations can be.

Traditional positional bargaining (eg, haggling over a price in a market) is a typical approach people take in negotiations. Although it is well known and works well for simple low-stakes transactions where ongoing relationships are not a priority, this approach is not optimal in ICEs. Interest-based negotiation (IBN) is a proven conflict management method that includes techniques and tools that serve as the foundation of many of the world’s preeminent conflict management groups [1,11]. This approach has been used in high-stakes negotiations where maintaining long-term relations is critical, such as the Israeli-Palestinian negotiation (Camp David Accords), Arias Peace Accords, and South African post-Apartheid constitutional reform [12,13]. Through this method, interests and goals are identified with the aim of working together to reach a mutually beneficial agreement. IBN has been used successfully for decades in multiple high-risk and high-stress environments and has been described as highly pragmatic, valuable, and effective by government, military, and Fortune 500 and Global 1000 leaders alike [14].

We believe that the techniques and tools that make up IBN can be learned [1,11]. Also, we believe that refresher training should be available to those in ICEs because when a high-stakes situation arises, crew members’ negotiation skills may have declined if they have not been used regularly. These hypotheses served as the impetus for creating this training module. A web-based, interactive-media approach was chosen to facilitate an active learning environment, which is easily accessible, self-directed, and more effective for performance and user satisfaction than traditional classroom or video learning alone [15]. A private, computerized format may also be more comfortable to users [3,16]. In ICEs, individuals need tools they can use on their own, not involving others, because the people they may need to negotiate with are part of their crew. Additionally, having a program that is accessible at any time allows people to learn or review these techniques when needed.

Intervention options in ICEs are minimal, and computer-based interventional programs applying cognitive behavioral instruction are promising [3]. Smith et al [16] recently reviewed work in this area and noted that advances in technology have led to several research programs focusing on digital technologies and countermeasures to support effective functioning of people in ICEs, though in many cases these have been driven by technological opportunity rather than cogent theory. They concluded that further studies and evidence-based interventions, particularly using well-being supportive design principles built to support individual autonomy, competence, and other key characteristics, were clearly needed [16]. Previous research on self-guided, interactive media–based negotiation training tools is limited, though a pilot study using electronic interactive-media content for problem-solving treatment showed that this approach was highly usable, acceptable, and credible [17]. In a separate study using this self-guided computerized cognitive behavioral tool to address issues of stress, depression, and conflict management, Guarino et al [18] showed that users found modules to be highly effective, easily usable, and acceptable. The attrition rate was high, however, as has been seen with other freely available web-based interventions [18].

In the IBN module, users are presented scenarios where they make choices and then get feedback based on their selections. The goal is making users aware of techniques to negotiate solutions while maximizing positive interpersonal relationships and accomplishing mission objectives. The program demonstrates how to solve important problems and address significant issues by creating sensible solutions the parties agree to, instead of reaching a standoff or a nonsensical compromise. The module provides a novel approach to learning vital conflict management skills, which is particularly relevant to people in ICEs, such as long-duration space missions, as well as for those who find themselves isolated in situations such as those created due to the COVID-19 pandemic. We hypothesized that individuals in ICEs would find the content acceptable, valuable for learning, and enjoyable.

## Methods

### Study Protocol

This was an opportunistic study rather than a formal trial or evaluation of this training tool. The module was provided to multiple individuals with a variety of backgrounds and in different countries, including a subset of 9 individuals in ICEs (6 from HI-SEAS [Hawai'i Space Exploration Analog and Simulation] Mars simulation [19] and 3 from Australian Antarctic Program wintering research stations). Additionally, several media articles were published during the COVID-19 pandemic specifically referencing this module as a tool to provide skills to combat stresses imposed through the COVID-19 pandemic and government-mandated stay-at-home orders; this led to an additional subset of users (n=21) who used the module during the pandemic. The module was open access so anyone from or in any country could participate, making this a convenience sample. Demographic or other personal information was not collected. The data in this study represent data collected from 2017 to 2020. Once users completed the module, there was a voluntary open survey that participants could complete from within the web module itself.

All participants received an identical questionnaire, and there was no randomization of items or adaptive questioning. The survey questions followed the same format and content used in previous studies with our interactive-media tools [17,18]. The survey included questions about realism, delivery, and value. No incentives were offered to complete the survey. All 10 numerical scaling questions and free-response questions were visible on the same web page. Participants were able to select only 1 response for each prompt, and they were unable to submit their responses to the questionnaire unless all numerical scaling questions were completed. Once participants submitted their responses, they were unable to review or change their answers. Cookies were not used to assign a unique user identifier to each client computer, and the IP address of the client computer was not used to identify potential duplicates. In rare cases with duplicate database entries being identified by same user ID, the first entry with all numerical scaling questions completed was used in analysis. No other techniques were used to analyze the log file for identification of multiple entries. As this study had many features of a web-based survey, we completed the CHERRIES (Checklist for Reporting Results of Internet E-Surveys) checklist to verify that the study met the requirements of a web-based survey study.

### Training Module

**Figure 1:**
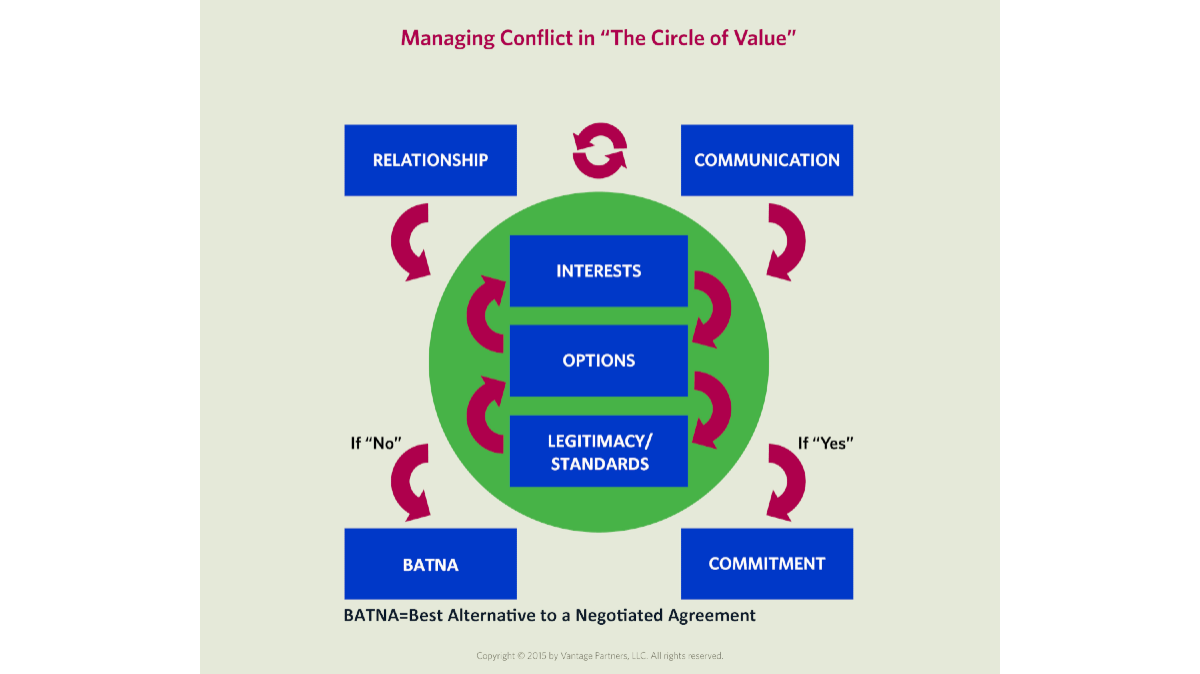
“The Circle of Value.” Reprinted with permission from Vantage Partners.

**Figure 2:**
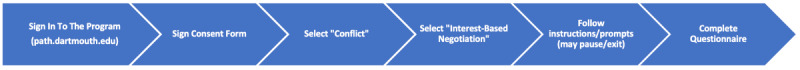
Overview of user flow.

The training module was scripted, filmed, and programmed to provide experiential training and scenario-based learning. The content highlights the core concepts of the IBN model for managing conflict as summarized in “The Circle of Value” (Figure 1). The module was designed to be completed as one session; however, participants could pause or even exit the program and return to their previous position at any time (Figure 2). The overall time to complete the module varies depending on participants’ selections and engagement, though by design the module can be completed in 45 to 120 minutes.

The module opens with a reference conflict scenario between a fictional International Space Station (ISS) crew and ground control. The scenario centers around an upcoming spacewalk (extravehicular activity [EVA]). The program’s videos provide the user with some background on the concerns and interests of both parties so the user can understand the context of the negotiation. The ISS crew believes that planning for a previous EVA was rushed and that the crew was blamed by ground control for the outcome. This has reduced trust among the crew in ground control and given them a desire to make sure that future events are fully planned. Ground control, on the other hand, wants to get a new spacewalk done quickly and is getting internal pressure to move fast. They think the previous problems were overblown and are the crew’s fault.

These interests (eg, the reasons why the crew wants to move slowly and why ground control wants to move fast), however, are not apparent to the parties shown in the negotiation. In the opening scenario, the negotiation follows a traditional positional bargaining model and goes poorly, with neither side satisfying their objectives. At this point in the module, negotiation expert and program mentor Jeff Weiss explains why the negotiation went poorly and introduces “The Circle of Value.” He briefly explains 3 of the main concepts of “The Circle of Value”: Interests, Options, and Legitimacy/Standards, and highlights the relevance of each to the opening scenario. He then gives 2 other real-world examples of the importance of viewing conflict through “The Circle of Value.” The user is then brought back to the opening conflict scenario for experiential training.

**Figure 3:**
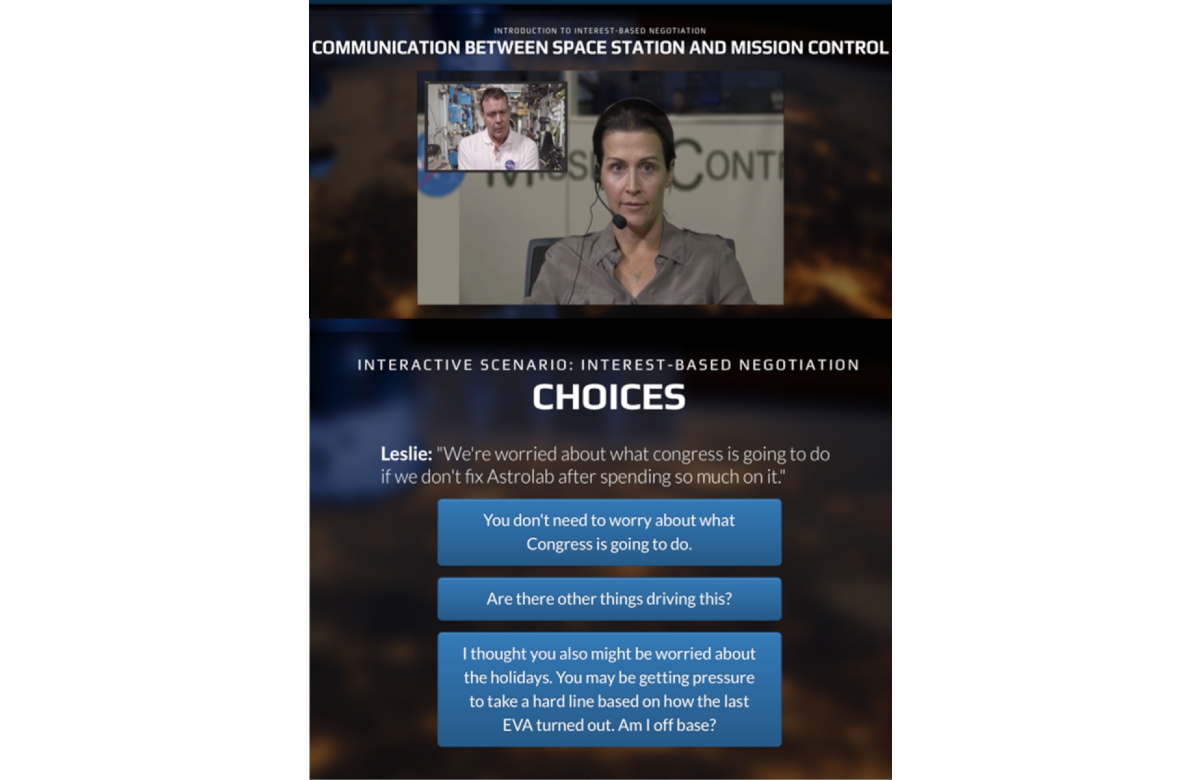
Scenario 2 with response choices. EVA: extravehicular activity.

In the next module segment, users move step by step through the interaction between the ISS crew and ground control. At each scene in the interaction where either ground control or the crew make a statement requiring a response, the user is presented with decision points. The user is asked to choose among 3 possible responses, each designed to facilitate a teaching point or demonstrate a particular negotiation technique (Figure 3). The choices offer a range of realistic responses including a “poor” response, an “acceptable” response, and an “optimal” response. Every selection prompts different feedback from the program mentor, and users are able to return to the beginning of the scene (and in some cases based on their choice must go back and choose another option) to explore feedback generated as a reaction to the various options offered.

The early decision points encourage the user to uncover interests (eg, why is the crew reluctant and why does ground control want to move quickly?), and the feedback highlights the importance of each party first taking the time to uncover the core objectives and concerns of the other party, rather than immediately staking out positions and starting to haggle. The importance of focusing on interests and not positions is emphasized, and the different response choices allow users to explore specific reactions to different language used to elicit these interests. Subsequent decision points instruct on how to develop options together and stress the importance of inventing ideas and continuing to brainstorm together, and not prematurely making a selection. The response choices for these decision points emphasize that this is a dynamic process and highlight that one can move back and forth between interests and options. The scenes and decision points then cover the importance of defining legitimacy and standards, which are objective criteria used to assess the options and determine which might be optimal (eg, if you reach a settlement, will all parties view it as fair based on established rules, experience, etc?). The program teaches a few ways to use these concepts and offers “rules of thumb” for use.

The scenarios also highlight common pitfalls in negotiating, such as failing to demonstrate active listening, as well as trying to address relationship issues by making substantive concessions. How to react to emotional responses is reviewed, along with techniques to reenter “The Circle of Value” by demonstrating understanding (but not necessarily agreeing with) the other person’s point of view. Ways of developing trust between parties are also discussed. The final section on choices exposes the user to the concept of the Best Alternative to a Negotiated Agreement (BATNA) and highlights when this should be used and in what capacity. For the purpose of evaluation, the module finishes with 3 free-response questions and 10 numerical scaling questions (0=strongly disagree to 4=strongly agree). The module is available on a web page [20].

### Analysis

Participants’ response selections for the short scenarios were tracked, as well as their free-text and numerical scaling answers. Data were analyzed for all participants who completed both the module and all the numerical scaling questions (n=54). Responses from participants who did not complete the numerical scaling questions were not included for analysis. Completion of the free-response section was not used as an exclusion criterion. Response selections were analyzed for distribution and trends by a population subset. Numerical scale questions were analyzed by mode (eg, the numerical response that was most frequent) across the different subsets, comparing both the 4 distinct populations of Non-ICE, HI-SEAS Mars simulation (HI-SEAS), Australian Antarctic Program (East Antarctica), and those who participated during the COVID-19 pandemic (Self-ID), and an aggregated traditional ICE subset (HI-SEAS and Antarctic) against Non-ICE and Self-ID participant responses.

### Ethics Approval

The Dartmouth College Committee for the Protection of Human Subjects (Dartmouth institutional review board CPHS 28777) approved this study protocol. Each participant provided consent before participating, and they were able to opt out of the study if desired. Study data were linked only to a user name chosen by the participant and later deidentified. Participation was voluntary, and no compensation was provided to participants. Each participant was presented with an electronic consent form before they could use the modules. The consent form described the purpose of the study, the estimated time to complete the modules, and who the investigator was. The participant was informed that the modules did not request any personal or identifying information and that the data were stored on secure servers.

## Results

**Figure 4:**
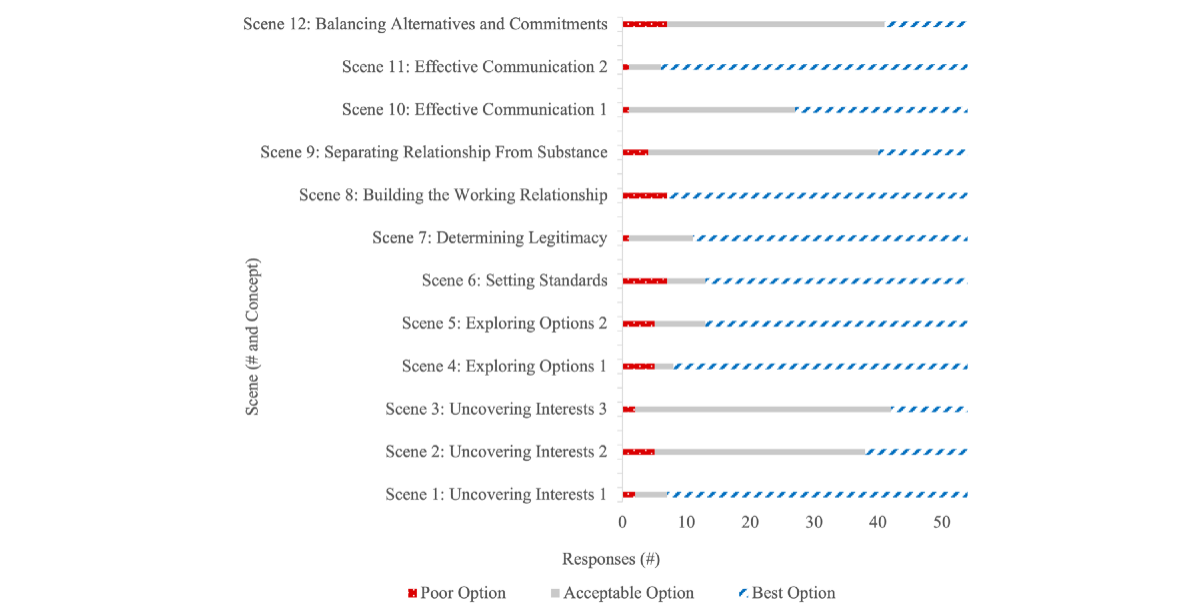
Variety and distribution within participant responses to each viewed scene.

The distribution of participant responses to each scene was varied (Figure 4). These data represent a participant’s first selected answer choice only and do not reflect whether a participant returned to the beginning of the scene and made an alternative selection. Excluding scene 8, all other scenes had at least 1 participant select each of the 3 responses. Of all 54 participants, 36 (67%) went back to the beginning of at least 1 scenario to make an alternative section. This included 12 of 24 (50%) from the Non-ICE subset, 3 of 3 (100%) from the Antarctic subset, 4 of 6 (66%) from the HI-SEAS subset, and 17 of 21 (81%) from the Self-ID subset. Of the 54 participants, 18 (33%) explored all 3 options of at least 1 scenario, including 3 of 24 (13%) from the Non-ICE subset, 2 of 3 (66%) from the Antarctic subset, 1 of 6 (17%) from the HI-SEAS subset, and 9 of 21 (43%) from the Self-ID subset.

**Figure 5:**
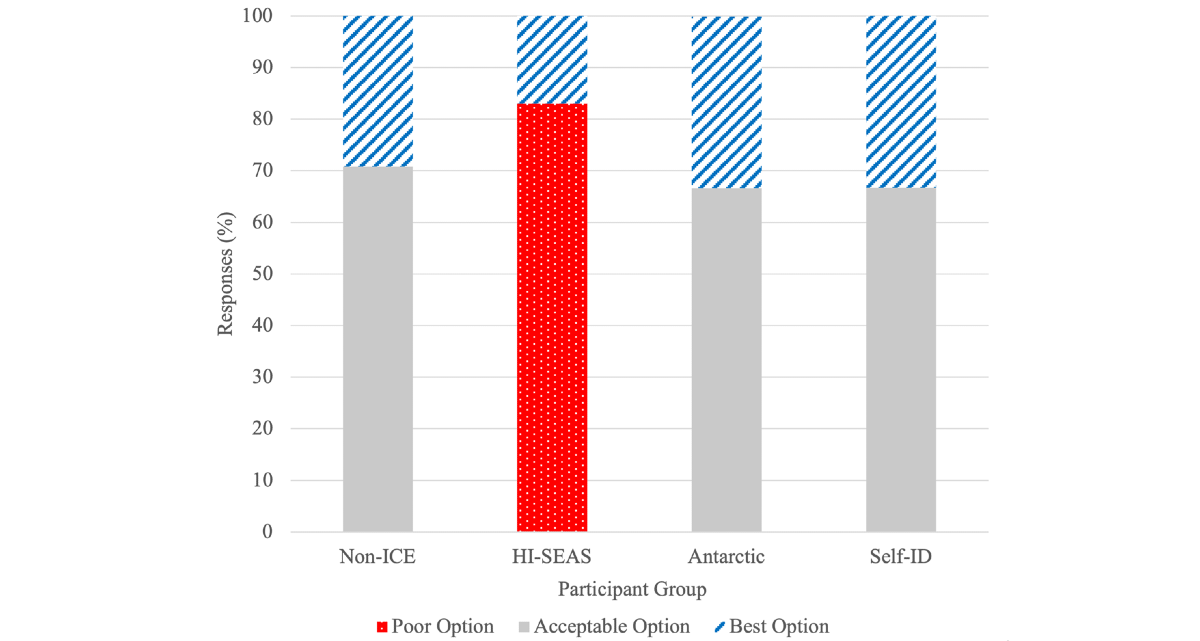
HI-SEAS inhabitant’s choices differed significantly from those of other groups in scenario 2. HI-SEAS: Hawai'i Space Exploration Analog and Simulation; Non-ICE: users who were not in isolated and confined environments; Self-ID: pandemic participants.

Answer selection varied across several scenes by a population subset (Figure 5). Figure 3 shows the 3 response options available in scenario 2 (Figure 4). The poor response option was not selected by any Non-ICE, Antarctic, or Self-ID participants, but was selected as the first response by 5 of 6 (83%) HI-SEAS participants. The acceptable option was selected by most of the Non-ICE (17/24, 71%), Antarctic (2/3, 67%), and Self-ID (14/21, 67%) participants, but by none of the HI-SEAS participants (0/6).

Numerical scaling responses to the 10 statements at the end of the module were analyzed by examining the cumulative number of scaled responses within each population subset (Table 1). A total of 51 of 54 (94%) participants found the activity valuable for learning about conflict management, including 9 of 9 (100%) ICE participants. The specific framework of “The Circle of Value” used in the module was felt to be valuable by 44 of 54 (81%) participants.

**Table 1:** Number of participants with particular reaction to statements.

Statements	Participants who strongly disagreed/somewhat disagreed/were neutral/somewhat agreed/strongly agreed^a^, n/n/n/n/n			
	Non-ICE^b^	HI-SEAS^c^	Antarctic	Self-ID^d^
The characters seemed like real astronauts and NASA^e^ employees.	2/0/7/*7*^f^/*8*	0/0/2/*3*/*1*	0/0/1/*2*/*0*	0/1/3/*13*/*4*
The scenario was like something that could actually happen on a real space mission.	0/0/6/*7*/*11*	0/0/0/*4*/*2*	0/0/0/*1*/*2*	0/0/4/*12*/*5*
The choices given in the scenario seemed like things a real crew member might have actually said or done.	0/0/2/*11*/*11*	0/0/1/*3*/*2*	0/0/0/*2*/*1*	0/1/4/*11*/*5*
Mr Weiss gave too much information in his spoken comments and advice during the interest-based negotiation activity.	*6*/*8*/4/5/1	*2*/*2*/1/1/0	*0*/*2*/1/0/0	*9*/*3*/5/2/2
The pace of Jeff Weiss' spoken comments and advice during the interest-based negotiation activity was too slow	*6*/*5*/5/6/2	*2*/*3*/1/0/0	*0*/*2*/1/0/0	*7*/*5*/6/3/0
I found “The Circle of Value” framework valuable.	0/1/5/*7*/*11*	0/0/0/*2*/*4*	0/0/2/*0*/*1*	0/0/2/*11*/*8*
Overall, I found the activity valuable for learning about conflict management.	0/0/1/*10*/*13*	0/0/0/*2*/*4*	0/0/0/*3*/*0*	0/0/2/*8*/*11*
Overall, I found doing the negotiation activity enjoyable.	1/1/4/*9*/*9*	0/0/2/*3*/*1*	0/0/0/*3*/*0*	0/2/3/*9*/*7*
The interest-based negotiation activity will probably be valuable for new members of a team in an isolated and confined environment.	0/0/2/*10*/*12*	0/0/1/*3*/*2*	0/0/1/*1*/*1*	0/0/4/*8*/*9*
The interest-based negotiation activity will probably be valuable for veteran members of teams deployed to an isolated and confined environment.	0/1/4/*10*/*9*	0/0/2/*3*/*1*	0/0/1/*1*/*1*	0/0/6/*6*/*9*

^a^Total number of participants=54 (24 Non-ICE, 6 HI-SEAS, 3 Antarctic, and 21 Self-ID).

^b^ICE: isolated and confined environment.

^c^HI-SEAS: Hawai'i Space Exploration Analog and Simulation.

^d^Self-ID: pandemic participants.

^e^NASA: National Aeronautics and Space Administration.

^f^Italicized values indicate positive responses to prompts.

The mode, or most common participant responses to these statements, was also calculated (Table 2). Most participants felt that the module would be valuable to new members of a team in an isolated and confined environment (46 of 54 participants, mode 4; 7 of 9 from the ICE subset, mode 3) as well as veteran members (40 of 54 participants, mode 3; 6 of 9 from the ICE subset, mode 3).

**Table 2:** Mode of responses to statements.^a^

Statements	Mode (ie, most common response)					
	All	Non-ICE^b^	HI-SEAS^c^	Antarctic	Self-ID^d^	
The characters seemed like real astronauts and NASA^e^ employees.	3	4	3	3	3	
The scenario was like something that could actually happen on a real space mission.	3	4	3	4	3	
The choices given in the scenario seemed like things a real crew member might have actually said or done.	3	4	3	3	3	
Mr Weiss gave too much information in his spoken comments and advice during the interest-based negotiation activity.	0	1	1	1	0	
The pace of Jeff Weiss' spoken comments and advice during the interest-based negotiation activity was too slow	0	0	1	1	0	
I found “The Circle of Value” framework valuable.	4	4	4	2	3	
Overall, I found the activity valuable for learning about conflict management.	4	4	4	3	4	
Overall, I found doing the negotiation activity enjoyable.	3	3	3	3	3	
The interest-based negotiation activity will probably be valuable for new members of a team in an isolated and confined environment.	4	4	3	N/A^f^	4	
The interest-based negotiation activity will probably be valuable for veteran members of teams deployed to an isolated and confined environment.	3	3	3	N/A	4	

^a^N=54 (24 non-ICE, 6 HI-SEAS, 3 Antarctic, and 21 Self-ID); 0=strongly disagree; 1=disagree, 2=neither agree nor disagree; 3=somewhat agree; 4=strongly agree.

^b^ICE: isolated and confined environment.

^c^HI-SEAS: Hawai'i Space Exploration Analog and Simulation.

^d^Self-ID: pandemic participants.

^e^NASA: National Aeronautics and Space Administration.

^f^N/A: not applicable.

## Discussion

### Principal Findings

Based on participant responses to the questionnaire statements (Tables 1 and 2), most participants across all settings found the activity realistic, valuable, and enjoyable. Specifically, participants found “The Circle of Value” approach valuable for learning about conflict management. A total of 128 of 162 responses (79%) indicated that participants found the module to be realistic, including 23 of 27 (85%) responses from ICE participants (Table 1). This was calculated by aggregating all “somewhat agree” and “strongly agree” responses for the first 3 statements. By absolute count, participants found the overall scenario itself and the dialogue or actions (statements 2 and 3) more realistic than the characters themselves (statement 1).

Interestingly, the distribution among the 3 answer choices was not equal. Although at first glance this might suggest that some participants did not identify as strongly with some of the responses, the specific distribution by participants in certain settings suggests that environment can play an important role (Figure 5). In scene 2, for example, response option 1 was highly favored by HI-SEAS participants and not selected by any of the other 3 subsets (Pearson chi-square *P*<.001). This particular response choice suggests that the other person’s concern might be misplaced (eg, “You don’t need to worry about…”). Although it is unclear exactly what aspect of HI-SEAS might be the cause of this response (mindset of people selected for HI-SEAS, environment of HI-SEAS, shared experience, etc), this finding suggests that people in certain environments may be drawn to different responses. Also, it suggests that responses to particular questions might be useful for exploring differences in individuals’ personalities, backgrounds, and experiences that might lead them to particular choices.

These responses demonstrate that further evaluation and expansion is worthwhile, particularly given the positive responsive from participants representing those in the specific target population of ICEs. The results indicate that this module provides valuable teaching tools in a way that participants found useful and realistic. The web-based format allows participants to complete the training independently, which is critical in ICEs as a person’s usual resources or support system might be the very people they need to negotiate with. In addition, when situations arise where conflict negotiation skills would be beneficial, people may not have recent experience with it or may want to revisit certain techniques and concepts now that they have a specific scenario they need to address. Given that conflicts can be mission terminating, people may want to enter these negotiations after having refreshed on the best way to do this effectively.

### Comparison With Prior Work

Managing conflict in isolated, confined environments and among deployed teams has been a focus for a long time within the military, NASA, and the corporate world. NASA uses several techniques to maintain psychological well-being on long-duration flights [6], including providing psychological support to astronauts and using National Outdoor Leadership School training. Many businesses use a combination of in-person or web-based didactics and team-building training. Although this module is not the first web-based training tool to have been developed or implemented [16], it is unique in the use of interactive media and the ability to offer an experiential rather than didactic training approach. It trains users in a well-tested approach to negotiation in a way that can be accessed remotely, done confidentially, and revisited for refresher training. We did not find any prior reference to a module specifically investigating conflict management through teaching IBN. Additionally, this study expands on previous work in this area by gathering data from individuals in actual isolated and confined environments, including a cohort that self-identified as isolated secondary to the COVID-19 pandemic. The particular advance with this module is providing training in a proven negotiation method that is engaging and can be accessed at the crew members’ convenience when needed.

### Strengths and Limitations

A key strength of this study is the incorporation of data from participants in several distinct ICEs. The module itself offers the ability to provide individuals anywhere with training in an effective approach to negotiation. Limitations of the study include a small sample size, particularly among participants in ICEs. By analyzing data only from those participants who completed both the module and the numerical scaling questions, a subset of participants are excluded, resulting in a selection bias. High attrition rates have been found with multiple freely available web-based interventions [18]. This subset could potentially include participants who exited because they did not find the module engaging or valuable. To help with this potential bias, a functionality has been added to the program to allow a participant to comment why they are exiting the program before completion. Nevertheless, all ICE participants who started the module completed the module and answered all numerical scaling questions.

This study was an opportunistic study and was not designed as a formal evaluation of this training tool. Minimal data on users were collected so demographic and background information on participants such as ethnicity, education, socioeconomic status, and location was not gathered. Such data could provide further insight into generalizability and help with a greater understanding of the subset of people actually using and completing the program. Although data gathered were limited, the data remain valuable because the tool was used by individuals in actual isolated and confined environments and so offers an insight into how people in this setting would respond to a tool like this.

### Future Directions

An important aspect of future work includes the addition of a follow-up questionnaire or prospective study to assess objectively whether the tools and skills presented in the training module are used by the participants and if this knowledge enhances the ability of participants to manage conflict successfully. In addition, understanding how participants use not only the skills presented but also the module itself (eg, reviewing before a conflict) can help guide development of future programs.

### Conclusions

The creation of this module demonstrates a novel, self-directed approach to providing vital conflict management skills. This web-based, interactive-media module for IBN training is realistic and well received by users who viewed it to be a valuable and enjoyable method for learning the concepts of IBN. The computerized format gives users the flexibility to use the program as needed, including for refresher training when conflicts arise. This study suggests that it is likely to be useful for both new and veteran individuals in isolated and confined environments, such as long-duration space missions, or those who self-identify as feeling isolated during the COVID-19 pandemic, as well as for anyone involved in a high-stakes negotiation where sustaining relationships is essential. Even when maintaining relationships is not critical, this approach addresses problems in a sensible way all parties can agree to. Previous research on self-guided, interactive media–based negotiation training tools is limited, which makes the research carried out here novel and shows that further evaluation and testing is worthwhile.

## Abbreviations

CHERRIES: Checklist for Reporting Results of Internet E-Surveys
EVA: extravehicular activity
HI-SEAS: Hawai'i Space Exploration Analog and Simulation
IBN: interest-based negotiation
ICE: isolated and confined environment
ISS: International Space Station
Self-ID: pandemic participants

## Data Availability

The data sets generated and analyzed during this study are available from the corresponding author on reasonable request.
